# Identification of a novel PTH1R variant in a family with primary failure of eruption

**DOI:** 10.1186/s12903-023-03226-1

**Published:** 2023-07-21

**Authors:** Yunchen Zha, Shushu Li, Yue-lin Yu, Zicheng Huang, Hai-ying Zhang, Weidong Kong

**Affiliations:** 1 School of Stomatology, Jinan University, Guangzhou, Guangdong China 510630; 2 Department of orthodontics, The First Affiliated Hospital of Jinan University, Guangzhou, Guangdong China; 3 Zhaoqing Medical College, Guangzhou, Guangdong, CA China

**Keywords:** Primary failure of eruption, *PTH1R*, New variant, Case report

## Abstract

**Background:**

Primary failure of tooth eruption (PFE) is a rare autosome genetic disorder that causes open bite. This work aimed to report a small family of PFE(OMIM: # 125,350) with a novel *PTH1R* variant. One of the patients has a rare clinical phenotype of the anterior tooth involved only.

**Case presentation:**

The proband was a 13-year-old young man with an incomplete eruption of the right upper anterior teeth, resulting in a significant open-bite. His left first molar partially erupted. Family history revealed that the proband’s 12-year-old brother and father also had teeth eruption disorders. Genetic testing found a novel *PTH1R* varia*nt* (NM_000316.3 c.1325-1336del), which has never been reported before. The diagnosis of PFE was based on clinical and radiographic characteristics and the result of genetic testing. Bioinformatic analysis predicted this variant would result in the truncation of the G protein-coupled receptor encoded by the *PTH1R*, affecting its structure and function.

**Conclusion:**

A novel *PTH1R* variant identified through whole-exome sequencing further expands the mutation spectrum of PFE. Patients in this family have different phenotypes, which reflects the characteristics of variable phenotypic expression of PFE.

## Background

Primary failure of eruption (PFE), proposed by Proffit in 1981, was defined as nonankylosed teeth failing to erupt fully or partially because of a malfunction of the eruption mechanism [1]. PFE is an autosomal dominant genetic disease influenced by genetic and environmental factors. It is associated with pathogenic mutations of *PTH1R*.

*PTH1R* is located on chromosome 3p21-p22.1 and is expressed in the kidney, lung, and oral organs [2]. *PTH1R* mutations can lead to several diseases: Jansen chondrodysplasia (MIM: #156,400), Blomstrand chondrodysplasia (BOCD, OMIM: #215,045), Eiken (OMIM: #600,002), and Ollier (OMIM: #166,000) disease [3]. PFE is the only disease associated with the haploinsufficiency of *PTH1R*, showing a phenotype only in the teeth and no defects in the skeleton of other body parts.

After the association between *PTH1R* and PFE has been found, more than 60 different *PTH1R* variants have been reported in PFE patients, including intronic, synonymous, missense, nonsense, and deletion variants. However, the location of the pathogenic *PTH1R* variant was observed scattered throughout the gene, with no domain accumulation, and no phenotype-dependent features were observed [4].

In this report, we made a thorough clinical examination and genetic testing of a small PFE family, identifying a novel pathogenic *PTH1R* variant (c.1325-1336del). Bioinformatic analysis showed that this variant causes truncation of the transmembrane region of the G protein-coupled receptor encoded by *PTH1R*, affecting the activation of the G protein.

This study was approved by the Ethics Committee of the First Affiliated Hospital of Jinan University. The procedures followed aligned with the 1975 Helsinki Declaration and its subsequent revisions. All patients signed an informed consent form before testing.

## Case presentation

### Clinical analysis of the proband

The proband was a 13-year-old adolescent from the First Affiliated Hospital of Jinan University whose right upper anterior tooth had not fully erupted, leading to an open-bite in the anterior region. The proband was above average in height (180 cm) and weight, with no craniofacial or skeletal dysmorphologies. He denied the habits such as tongue thrusting” and finger sucking.

**Fig. 1:**
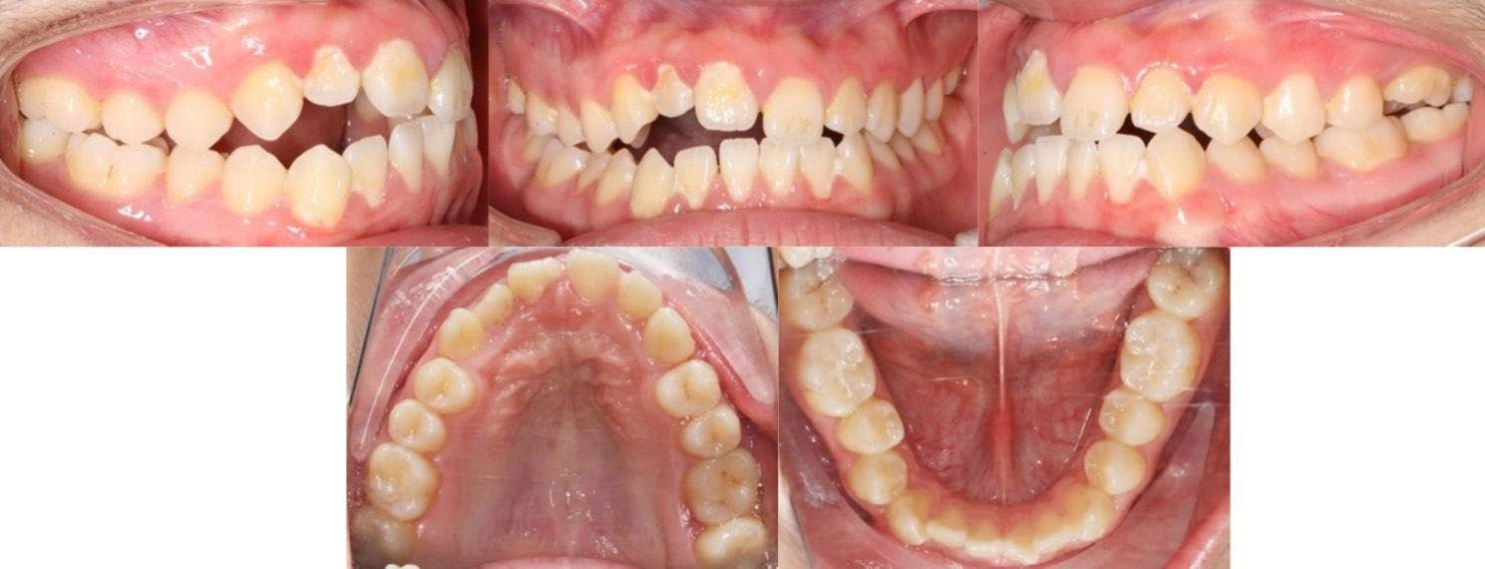
Pretreatment intraoral photographs

The clinical examination revealed that the right maxillary anterior teeth of the proband [11–13] were below the occlusion plane, forming an open-bite of 4 mm. The eruption height of the left maxillary first molar (26) was slightly insufficient. Bilateral molars were of a complete mesial relationship (Fig. 1). All the impacted teeth had no caries or enamel defects, and the position of the adjacent teeth was normal. There were no other apparent physical abnormalities in all four quadrants.

**Fig. 2:**
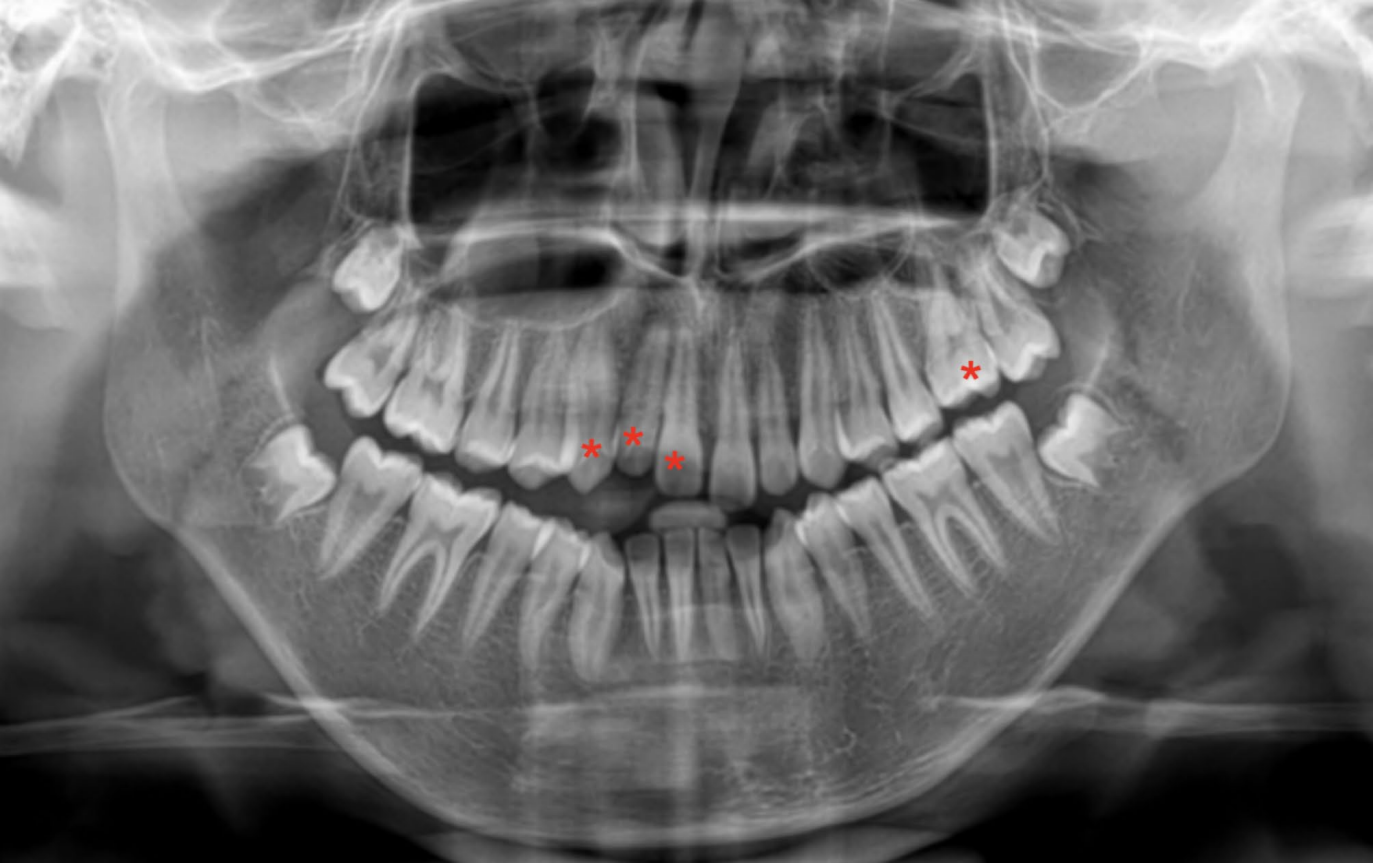
Pretreatment panoramic radiograph

**Fig. 3:**
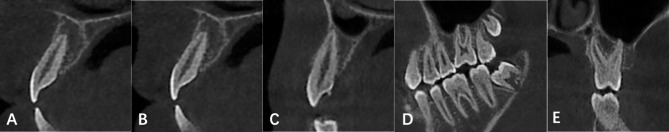
CBCT images of impacted teeth before treatment(**A**, the sagittal image of #11. **B**, the sagittal image of #12. **C**, the sagittal image of #13. **D**, the sagittal image of #26. **E**, the coronal image of #26.)

A panoramic radiograph revealed that the impacted teeth’ roots were well-developed, with no physical barriers such as dental tumors, cysts, etc. (Fig. 2). CBCT showed no radiolucencies around the root apices of the #11,#12,#13,#26. The periodontal membrane space is clear and complete, with no signs of ankylosis (Fig. 3). The normal percussion result further ruled out the possibility of ankylosis.

**Fig. 4:**
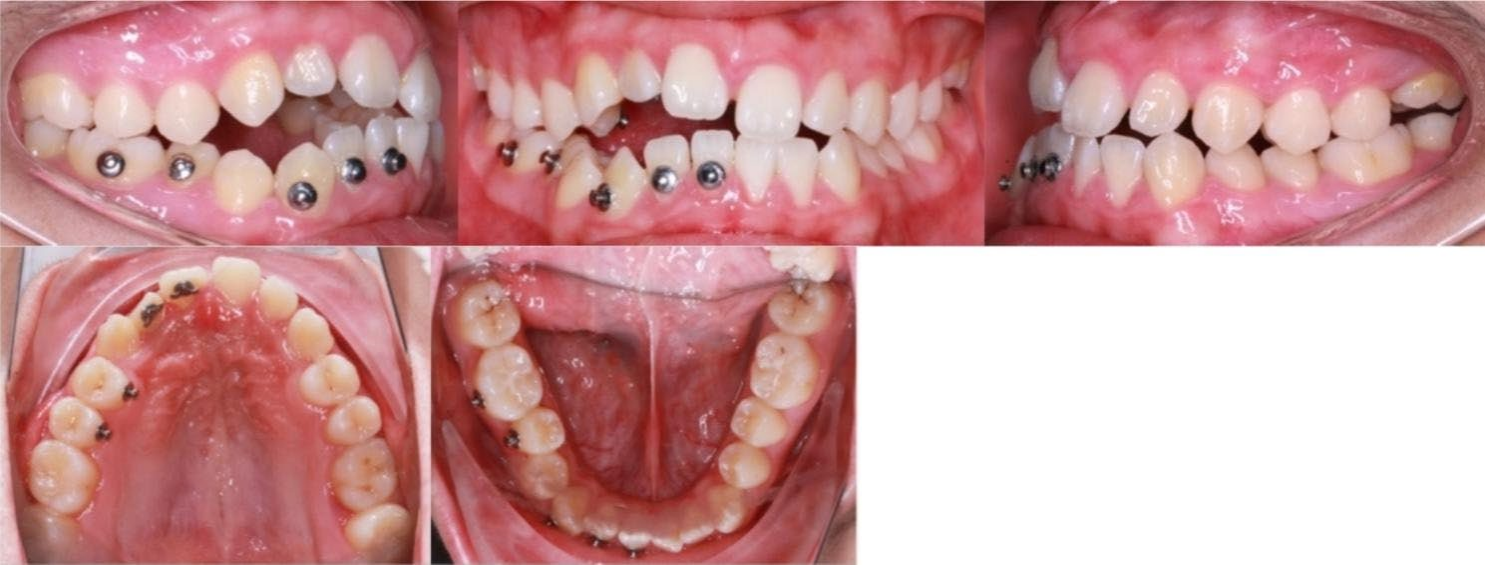
Intraoral photographs during treatment

The proband was still in the peak of growth at the first visit; after a year of observation, it was found that the open bite was increasing. We made local traction to the impacted teeth. Bonding lingual button at the palatal surface of #11,#12,#14,#15 and the labial surface of #41,#42,#43,#45,#46 (Fig. 4). It was followed by vertical intermaxillary traction with rubber bands between the upper and lower buttons.

**Fig. 5:**
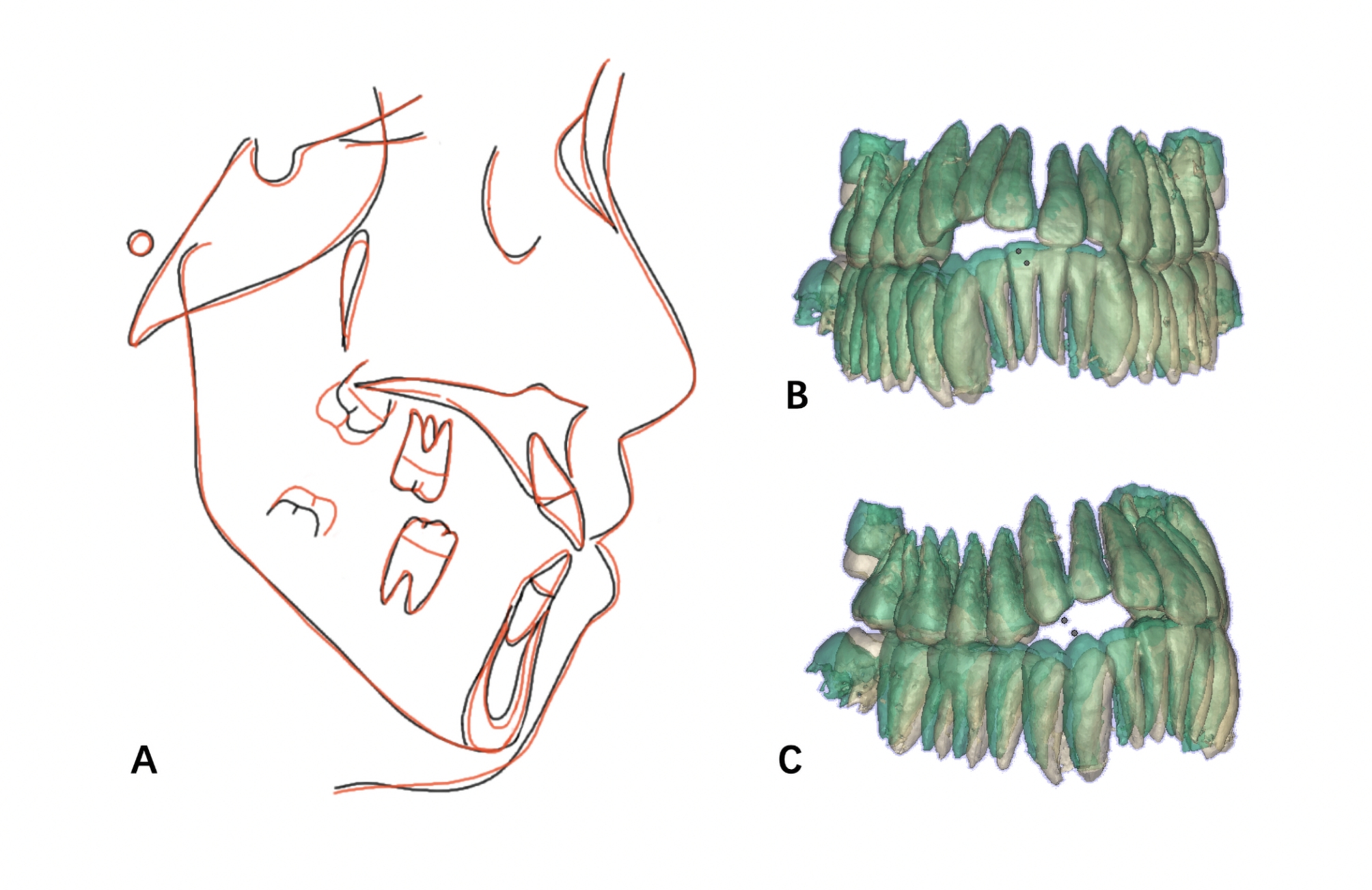
**A**, Initial (black), and final (red) cephalometric tracings are superimposed on the anterior cranial base. **B** and **C**, Initial (green) and final (yellow) 3D models of tracings are superimposed

**Fig. 6:**
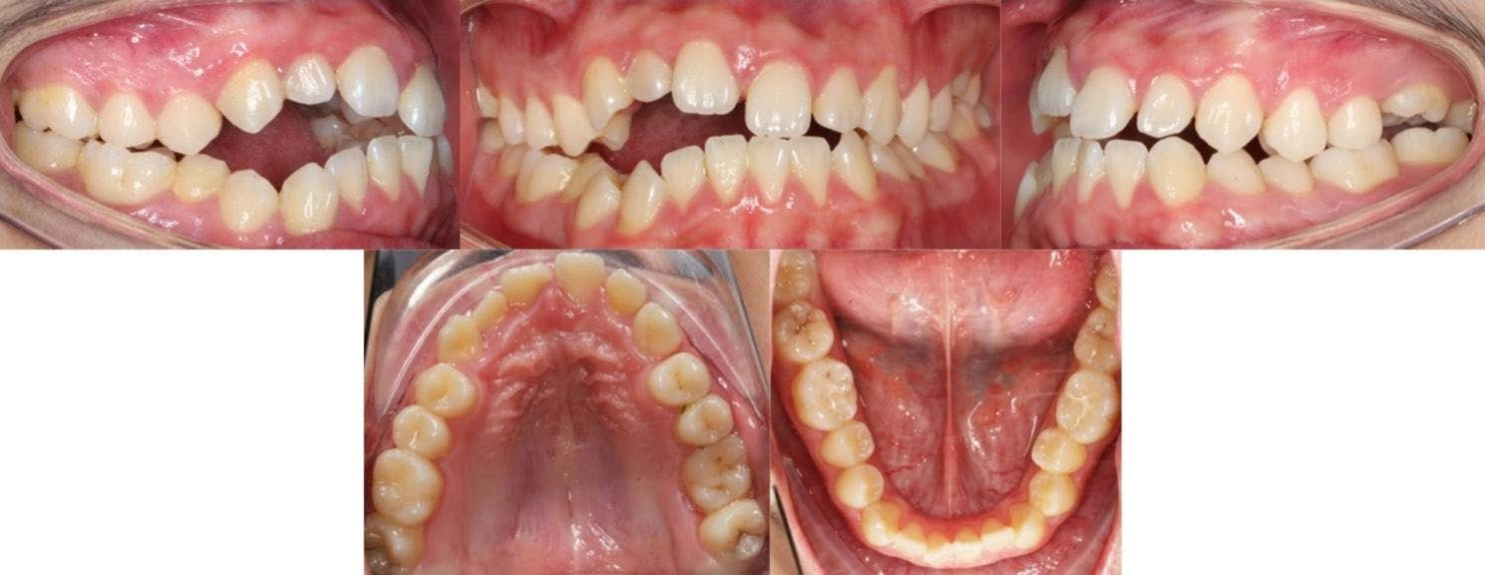
Intraoral photographs of proband at one year recall

After six months of traction, the open-bite of the anterior tooth area did not reduce. We used Mimics 15.0 to convert CBCT data into 3D models before and after orthodontic treatment and superimposed them to evaluate the three-dimensional movement of the impacted teeth. The results showed that only the third molars grew, while the anterior teeth barely moved (Fig. 5). At one year recall, we found that the degree and range of open-bite in the anterior and posterior dental areas further increased (Fig. 6).

### Clinical analysis of other family members

**Fig. 7:**
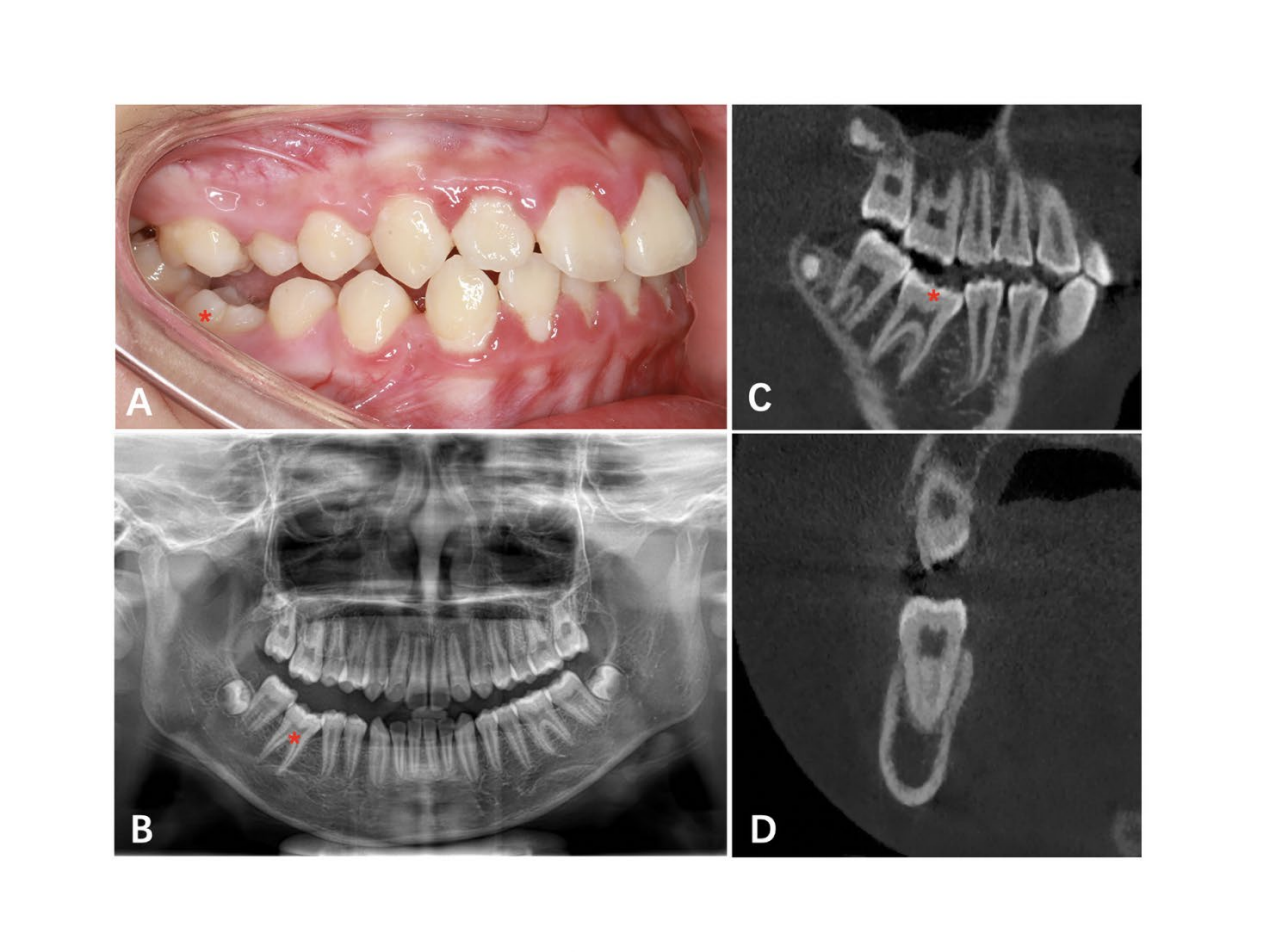
Clinical and radiographic information of the proband’ bother. (**A**, intraoral left view. **B**, panoramic radiograph. **C**, sagittal image of #46. **D**, coronal image of #46. “*” represent the PFE involved tooth. )

The proband’s brother was 12 years old, whose right mandibular first molar(46) was below the occlusion plane, but the adjacent teeth erupted to average height (Fig. 7A). Panoramic radiograph and CBCT showed normal root morphology and no periapical inflammation of #46 (Fig. 7B, C, D). The open-bite aggravated after a year of observation. As the growth and development proceed, the teeth adjacent to #46 and the surrounding soft tissue of #46 grow normally, causing it submerge.

**Fig. 8:**
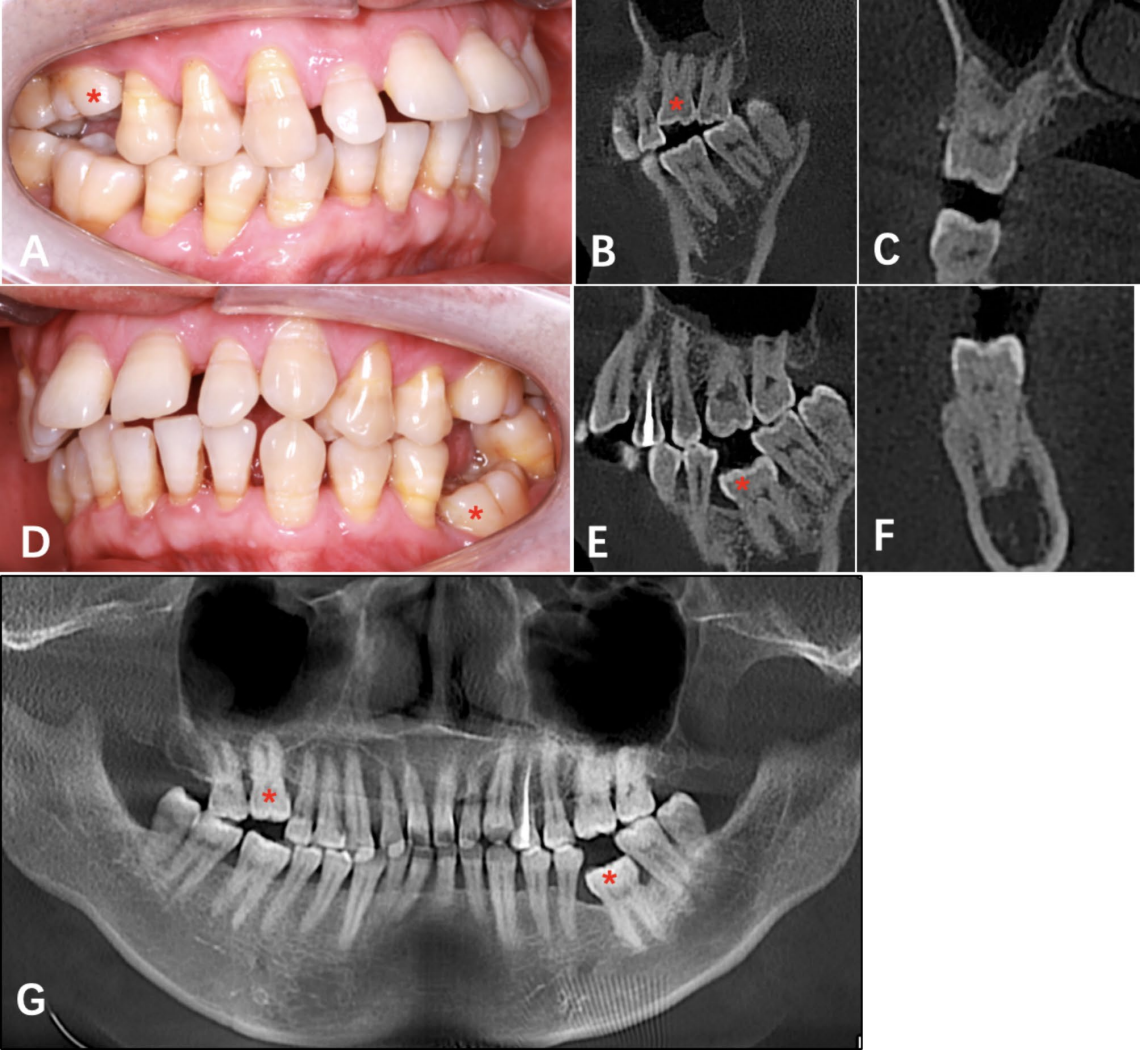
Clinical and radiographic information of the proband’ father. (**A**, intraoral right view. **B**, the sagittal image of 16. **C**, the coronal image of 16. **D**, intraoral left view. **E**, the sagittal image of #36. **F**, the coronal image of #36. **G**, panoramic radiograph. “*” represent the PFE involved tooth.)

The proband’s father also had two impacted teeth in the dentition. The #16 and #36 were below the occlusion plane leading to a 3 mm open-bite on the right and a 6 mm open-bite on the left (Fig. 8A). Alveolar bone resorption indicates chronic periodontitis in this patient. Radiographic information showed that the morphology of #16 and #36 was normal, the roots were well developed, and there were no abnormalities in periapical tissue (Fig. 8B-G).

### Genetic analysis

#### DNA extraction and sequencing

We sequenced the *PTH1R* gene sequence of the proband and his relatives. DNA was isolated from 4 to 6 ml EDTA blood samples using a standard desalting procedure. NCBI Genebank was applied to identify the sequence information of the PTH1R gene and use Primer premier 5.0 software to design 11 pairs of primers. Table 1 showed the primers used for sequencing the coding sequence of the *PTH1R* gene and exon-intron connection. The exons of the *PTH1R* gene were amplified by polymerase chain reaction (PCR). The exons of the *PTH1R* gene were pre-denatured at 94 ℃ for 5 min, denatured at 94 ℃ for 30 s, annealed at 55–60 ℃ for 35 s, extended at 72 ℃ for 10 min, and cycled for 35 times. PCR single primer was used as a sequencing primer for the sequencing reaction. NM_000316.2 was used as the reference sequence of the *PTH1R* gene. Identified variations were confirmed in a new PCR and sequencing reaction. Finally, the sequencing results were compared with the *PTH1R*-based standard sequence in Genebank.

**Table 1:** Primer information for *PTH1R*

Exon	Forward primer(5’-3’)	Downstream primer(3’-5’)	PCR product lengths(bp)
**Exon1**	ATCTGAACACCGGCACACTT	ACAGATGTGTCTCCATGCGG	417
**Exon2**	ACGCTGGAGCTCTAAGAGGA	ACGCTGGAGCTCTAAGAGGA	392
**Exon3**	GATTTCTTCGCTCCGAGGCA	TCGAACTACTGGGGGAACTCA	497
**Exon4**	CAGCCTACAGGGTTACTGGC	CCTTTACCTAAGTCCGCCCC	491
**Exon5**	CCCACTAGCTGATCCTGCAC	TATAAGAGCCAAGAAGCATGAGC	494
**Exon6-7**	ACAAACCACAGATGTATTCATCCTT	GAGCATCAGGGTAAGAGCAAGAG	567
**Exon8-9**	CTGACCCCTGACCTTGACTCCTC	TATAAGACGGGTTTGAGTGGCTGAA	730
**Exon10**	CGGCACCACTTGTCCTCTC	AGCCTGGAATAGGGTCAGGA	419
**Exon11-13**	GAAGCTGTTAGGGCACCACA	CCCCCTGCAACCAACTCTTT	1299
**Exon14-15**	GTTGTCCTCCCATGGTGACT	ATGTCCTCAGGGGTGTTCTTG	527
**Exon16**	TCCCAGGAGTCCCCTATTCC	GTGACCCCTGCAGACTTGAG	837

**Fig. 9:**
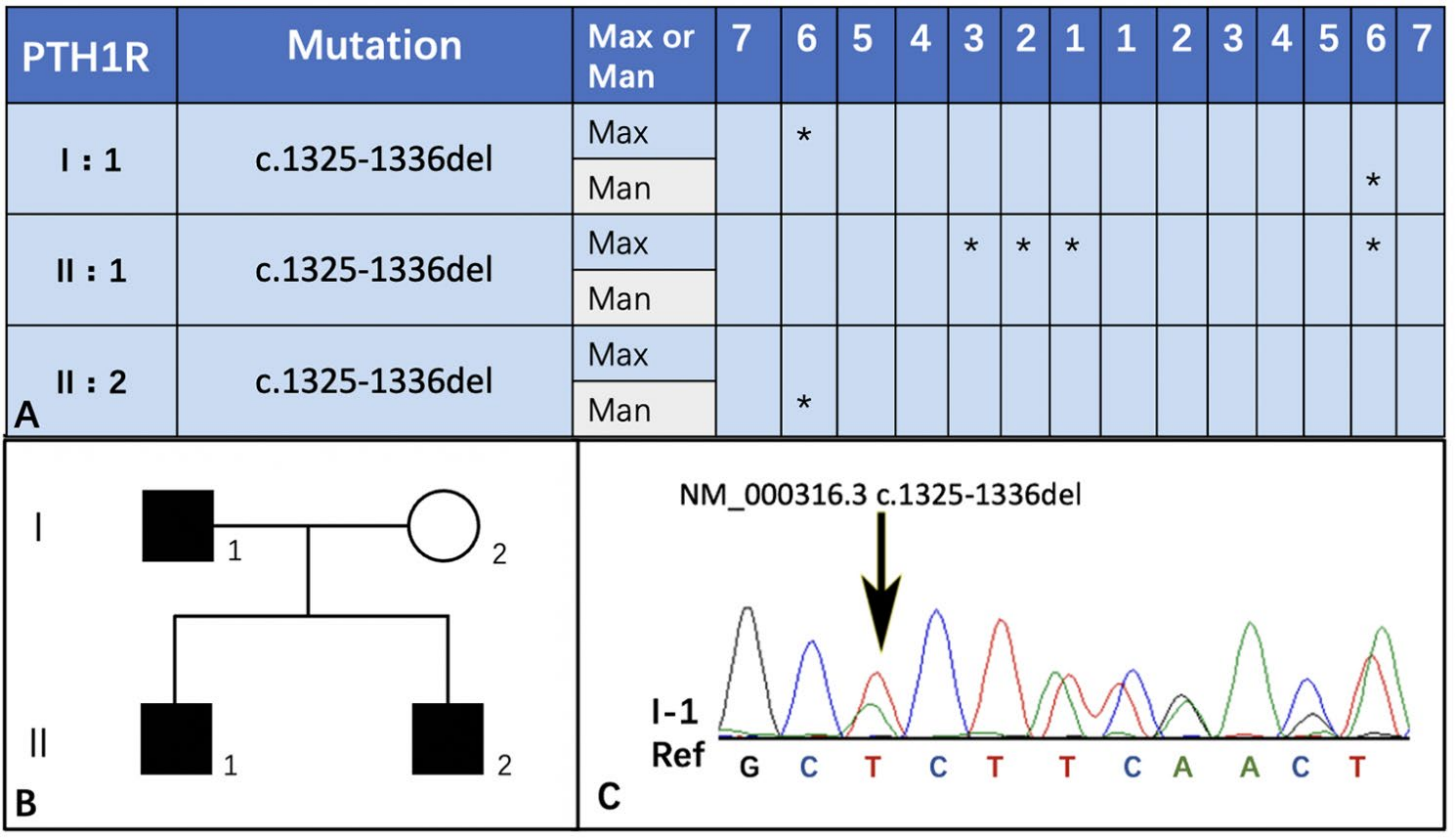
Phenotype and genetic testing results. (**A**, PFE-involved teeth position of the PFE patients in this family. I:1 represent the father, I:2 represent the mother, II:1 represent the brother of the proband, and II:2 represent the proband. **B**, pedigree of this family. **C**, representative electropherogram of the *PTH1R* sequence segregating. A double peak occurred at the black arrow, indicating that there is a normal single strand of the *PTH1R* gene and a mutated one. The mutations of three patients are at the same site of the electropherogram.)

The results of Sanger sequencing showed that the genotype of the proband’s mother (I-2) was normal, and other patients (I-1, II-1, II-2) had *PTH1R* variant (c.1325-1336del). Therefore, the *PTH1R* gene mutation in this family was derived from the father (Fig. 9). Sequencing analysis by Contig revealed this variant would cause the deletion of the “ACTATGAGATGC” in the exon 14 of the *PTH1R* gene. The missing amino acids are methionine, histidine, tyrosine, and glutamic acid. This variant was named NM_000316.3 c.1325-1336del, which has never been reported in association with PFE and is not present in the Genebank and ExAC databases.

### Bioinformatic analysis

**Fig. 10:**
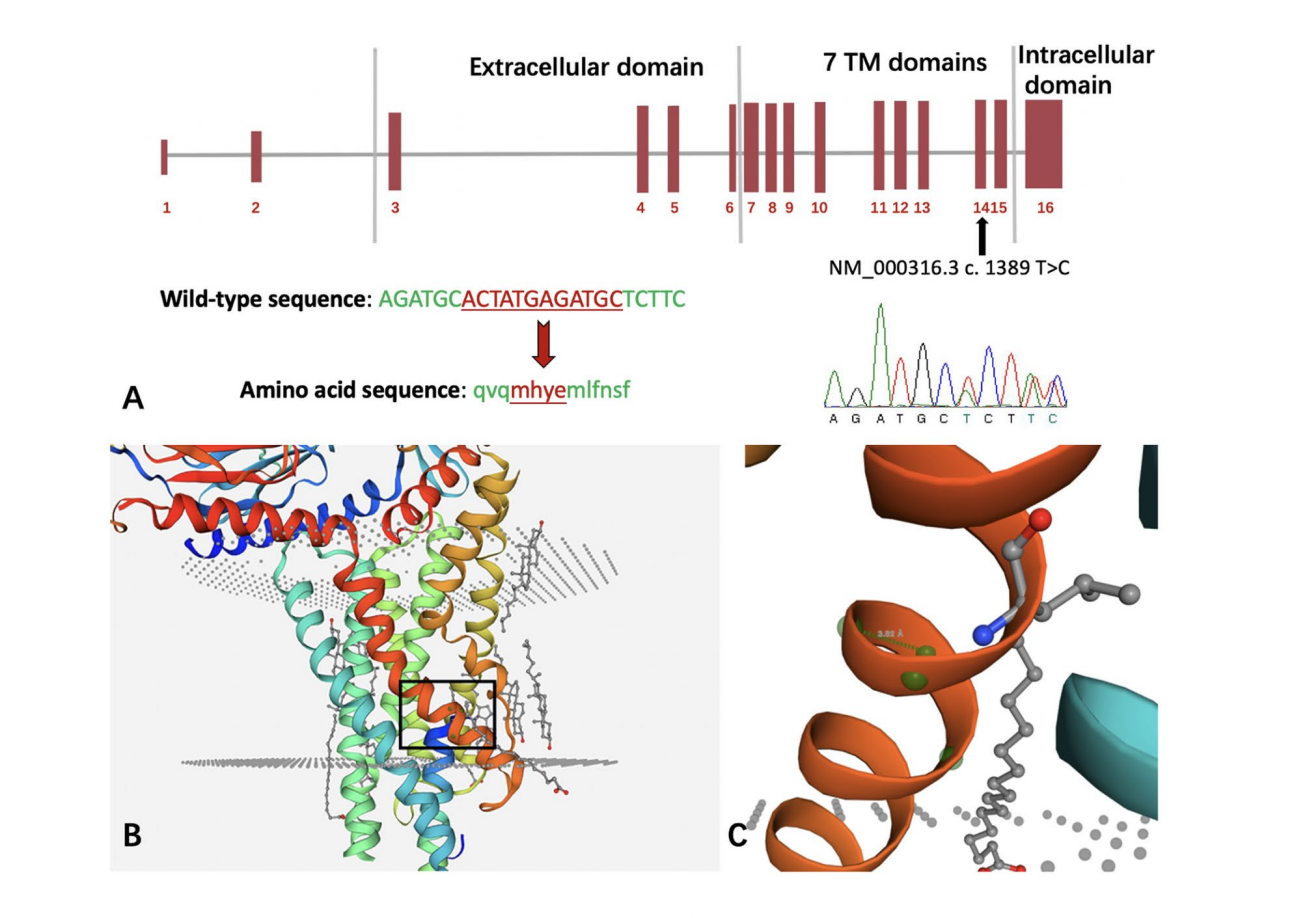
**A**, the distribution of PPR exons and the position of c.1325-1336del. The normal sequence and corresponding amino acid sequence of the gene are in the left corner. The red part shows the missing segment. **B**, the 3D structure of PPR which contain 7 segments ofαhelices embedded between the lipid bilayer. The variant occurs in the red transmembrane region indicated by the black box. **C**, the specific location of the variant on the helix and the green globules represent the missing amino acids

PTH1R encodes the Recombinant Parathyroid Hormone receptor Receptor1 (PTHR1 or PPR). Compare the CDS sequence of the *PTH1R* gene and the amino acid sequence on the Ensembl genome browser website (http://asia.ensembl.org/index.html). It showed that the missing part of the gene encodes 441–444 amino acids, located in the seventh segment transmembrane region of the PPR (44–463). Using the Mutation tasing (https://www.mutationtaster.org/MT69/MutationTaster69.cgi) to analyze the Pathogenic mechanism of c.1325-1336del, this variant results in the loss of the seventh transmembrane of the PPR and the downstream structures binding to the G protein. Made the interaction with G protein subunits GNB1 and GNG2 significantly reduced. And the PPR lacks the sites binding to the G protein (Table 2). The variant’s position on the PPR protein structure is shown using the SWISS-MODEL to build the protein structure model after Mutation (https://swissmodel.expasy.org) (Fig. 10).

**Table 2:** Structural and functional changes in the PPR protein upon mutation

start (aa)	end (aa)	feature	details	
441	463	TRANSMEM	Helical; Name = 7; (Potential).	lost
464	593	TOPO_DOM	Cytoplasmic (Potential).	might get lost (downstream of altered splice site)
471	471	CONFLICT	K -> N (in Ref.2).	might get lost (downstream of altered splice site)
473	473	CONFLICT	S -> C (in Ref.2).	might get lost (downstream of altered splice site)
474	474	MUTAGEN	W->A: Strongly reduced interaction with G protein subunits GNB1 and GNG2; when associated with A-477.	might get lost (downstream of altered splice site)
474	477	MOTIF	Important for interaction with G proteins.	might get lost (downstream of altered splice site)
477	477	MUTAGEN	W->A: Strongly reduced interaction with G protein subunits GNB1 and GNG2; when associated with A-474.	might get lost (downstream of altered splice site)

## Discussion

The clinical manifestations of PFE are easily confused with other types of eruption disorders, and a precise diagnosis is required before orthodontic treatment. The six elements of PFE diagnosis are : (1) PFE primarily affects posterior teeth, especially the first molar; (2) teeth distal to a PFE-affected molar are always affected, causing posterior open bite; (3) eruption pathway of the involved tooth is clear; (4) the vertical alveolar process of the involved tooth were significantly lower than the adjacent teeth. (5) lead to an open-bite; (6) deciduous teeth in the mixed dentition can be involved [4–6]. However, the phenotypes of PFE are diverse, with differences in clinical manifestations between different families or patients in the same family.

In this report, the eruption obstacle of the proband mainly occurred in the anterior tooth area. The proband’s father and bother’s mesial teeth erupted to average height. These symptoms differ from the six elements of PFE described above and are easily misdiagnosed with mechanical eruption failure (MFE). The keys to distinguishing between PFE and MFE are as follows [7, 8]. (1) PFE has no mechanical obstacle factors, while MFE is caused by physical barriers that can be found clinically, such as retention of deciduous teeth, dental tumors, cysts, and so on. (2) PFE patients have familial aggregation, while MFE is sporadic. (3) Teeth distal to a PFE-affected molar are usually affected, while MFE presents with one molar involvement. (4) PFE patients are often accompanied by skeletal class III malocclusion, while MFE patients have no specific type of malocclusion. (5) The MFE teeth have metallic sounds when percussion, while PFE teeth have not. PFE and MFE can be distinguished according to the above indicators. But when encountering more complicated situations, we need to confirm the diagnosis by genetic testing.

Genetic testing is an effective method for diagnosing PFE. To date, 64 *PTH1R* variants associated with PFE have been reported. The novel variant in our report further expands the mutation spectrum of PFE. *PTH1R* encodes the parathyroid hormone (PTH) receptor type 1 (PTH1R), a Class B G protein-coupled receptor (GPCR) with seven transmembrane spanning helixes. Binding to PTH or PTH-related protein (PTHrP), PTH1R activates two major second messenger signaling systems, including the adenylyl cyclase/protein kinase A pathway and the phospholipase C/protein kinase C pathway [9]. The PTHrP-PTH1R pathway regulates tooth eruption in multiple aspects. PTHrP-PPR directly regulates the proliferation of dental mesenchymal progenitors and their differentiation into cementogenic cells, periodontal membrane cells, and alveolar bone osteoblasts. PTHrP-PPR indirectly regulates the bone resorption activity of osteoclasts through the RANKL-RANK pathway [10, 11]. The autocrine signaling of PTHrP-PPR participates in the main signaling pathways Wnt/β-catenin, Hh, and TGF-β/ BMP. It affects tooth eruption and root formation by regulating cementum formation and periodontal membrane attachment [11].

Previous studies have confirmed that the pathogenic mechanism of *PTH1R* gene mutation is related to the truncation of the PPR protein resulting in decreased signal transduction function. Hendricks found that (c.1092delG) caused the absence of the second and third extracellular loops responsible for ligand recognition and interaction on the PPR protein structure, causing the cells lack effective G protein interactions [12]. Subramanian found that (c.1016G > A) affected the signal transduction function of G proteins by misfolding or degradation of the protein [13].

Genetic analysis of our patients revealed a new variant in exon 14 of the *PTH1R* (c.1325-1336del) that introduces a deletion. Moreover, it likely produces a truncated protein lacking seventh transmembrane domains and the critical downstream structure for binding to the G protein, causing the interaction of the G protein subunits GNB1 and GNG2 to be significantly reduced, resulting in the lack of sites binding to the G protein. This variant can be pathogenic as it causes conformational changes in PPR, leading to dysfunction in signal transduction and subsequently affecting downstream pathways mediated osteogenesis and osteoclast function or the development of the periodontal tissues, which are the keys to tooth eruption.

Among the identified *PTH1R* mutations, c.1324 C > G, c.1355G > A, c.1348-1350del, c.1389 T > C are also located in the seventh transmembrane region of the PPR [5, 14]. Nonsense variants and deletion variants causing a frameshift (c.1324 C > G, c.1355G > A, c.1348-1350del) are pathogenic because they cause the partial deletion of the seventh segment transmembrane structure of PPR protein and cause the protein structure gain of the helix, loss of strand or altered ordered interface. The synonymous variant (c.1389 T > C) does not cause amino acid alteration, but it can alter the efficiency of mRNA splicing, changing the structure and function of the protein [15]. The intron mutations occurring around the seventh transmembrane region (c.1353-1G > A, c.1354-1G > A) also led to the family PFE. The pathogenic mechanism is completing the skipping of exon 15 [16].

Except for the proband, other members of this family mainly involved posterior teeth. Variable expressivity between affected family members was noted. The reason for this phenomenon is diverse, with the primary factor being that both epigenetic and microenvironmental influences can cause PFE. Growth environment, nutrient level, maternal body, and ontogeny can affect protein modification, such as DNA methylation and acetylation of histones. The PPR protein encoded by the *PTH1R* gene experiences a modification process of phosphorylation and glycosylation post-translationally. Therefore, different environmental factors during individual growth and development can lead to different protein modification levels, ultimately determining the phenotypic differences between the patients.

Second, PFE patients carry a normal copy of the *PTH1R* gene and a mutated one. At the same time, the severity of the disease is affected by the expression level of the regular portion of *PTH1R*. The higher the expression level of the normal allele, the greater the expression of PPR. In contrast, low expression causes insufficient PPR dosage, results in a more severe PFE phenotype, and even develops a systemic disease, such as BOCD. Therefore, increased expression of the normal *PTH1R* allele may ameliorate pathology and explain some of the highly variable expressivity of PFE.

Finally, DNA sequence analysis was explicitly directed towards the coding and immediately flanking intronic sequences of the *PTH1R* gene. Deep intronic regions and regulatory sequences of the gene may have been missed. The undetection of the missed intronic sequence may explain a difference in phenotype [14]. Moreover, not all PFE patients carry the *PTH1R* variant, indicating that unidentified genes may also be responsible for PFE. These potential pathogenic genes may regulate part of the PFE phenotype.

## Conclusion

We reported a family in which all PFE patients carry the *PTH1R* variant (c.1325-1336del). The clinical phenotype of PFE varied among pedigree members, suggesting that PFE is a genetic disease with variable expressivity. The pathogenic effect of c.1325-1336del may be related to the truncation of the seventh transmembrane region of the PPR protein and the downstream protein structure, leading to a decrease in signal transduction function.

## Data Availability

DNA of family members and original sequence electropherograms are available upon request. The accession number of this new variant in ClinVar is SCV003915837.

## Abbreviations

PFE: Primary failure of eruption
PCR: Polymerase Chain Reaction
PTH1R: Parathyroid Hormone 1 Receptor
